# Reply to Musculus et al.: A case of offside in scientific discourse?

**DOI:** 10.1073/pnas.2507207122

**Published:** 2025-06-06

**Authors:** Leonardo Bonetti, Torbjörn Vestberg, Reza Jafari, Debora Seghezzi, Martin Ingvar, Morten L. Kringelbach, Alberto Filgueiras, Predrag Petrovic

**Affiliations:** ^a^Center for Music in the Brain, Department of Clinical Medicine, Aarhus University and The Royal Academy of Music, Aarhus, Aalborg 8000, Denmark; ^b^Centre for Eudaimonia and Human Flourishing, Linacre College, University of Oxford, Oxford OX39BX, United Kingdom; ^c^Department of Psychiatry, University of Oxford, Oxford OX37JX, United Kingdom; ^d^Department of Psychology, University of Bologna, Bologna 40127, Italy; ^e^Center for Psychiatry Research and Center for Cognitiveand Computational Neuropsychiatry, Department of Clinical Neuroscience, Karolinska Institutet, Stockholm 17177, Sweden; ^f^College of Psychology, School of Health and Applied Sciences, Central Queensland University, Cairns 40939, Australia; ^g^Department of Psychology, School of Education and Social Sciences, Rio de Janeiro State University, Riode, Janeiro 24435-000, Brazil

We appreciate the opportunity to respond to Musculus et al.’s (1) letter regarding our recent study on elite soccer players and cognition (2). While we welcome scientific discourse, we believe that many of their comments are unfounded or misrepresent our findings and methodology.

First, Musculus et al. argue that elite soccer players should have been compared to lower-level players rather than a general population control group. However, this was not the aim of our study. Previous research has already demonstrated that elite players exhibit significantly better executive functions (EF) than subelite players (3) and that those who have competed at the national level outperform those who have not (4). Instead, our objective was to replicate previous findings of markedly superior cognitive abilities in elite players using a much larger sample and a larger set of cognitive tests than previously, addressing the limitations of prior studies, which suffered from small sample sizes, low statistical power, and a narrow focus. Our results confirmed this pattern, as our study showed that elite players largely outperformed controls and the norm.

Second, although in the original manuscript we argue that previous knowledge seems to point toward a causal relationship between cognitive abilities and elite soccer performance, we do not claim to show a causal relationship in our data. We also emphasize the importance of future longitudinal research to further explore potential predictive relationships.

Third, Musculus et al. suggest that we overlooked other relevant factors in talent identification. We fully agree that talent identification is multifaceted, involving technical, tactical, psychosocial, and physical attributes. However, acknowledging the importance of additional variables does not negate the strong significance of the psychological attributes we examined.

Fourth, the assertion of overfitting in our machine learning analyses is unsubstantiated. As clearly outlined in our paper, we implemented a well-known permutation-based cross-validation approach specifically to prevent overfitting and ensure robustness. These are standard techniques in machine learning and effectively mitigate the issue raised by Musculus et al. Additionally, our results were corroborated by traditional statistical analyses, reinforcing their validity.

Finally, Musculus et al. (1) claim that our conclusions contradict existing literature. However, a substantial body of research has established that EF are associated with elite performance in soccer and other ball sports, questioning the validity of the claim of Musculus et al. (1). Moreover, their assertion that sport-specific tests are better predictors of future success (5) is irrelevant, as it misses the core focus of our study. While previous research, including our own, has demonstrated that EF relate to both performance and the prediction of elite play (3, 4, 6–8), as well as coach-rated game intelligence (4, 7), our primary objective was not prediction. Rather, our study aimed to deepen the understanding of the cognitive processes that underpin behavior in the context of soccer.

In conclusion, while we acknowledge that more cognitive and behavioral neuroscience is needed in the field of sport psychology, we stand by the validity of our study’s findings, claims, and interpretations.
